# FLDS: A Comprehensive dsRNA Sequencing Method for Intracellular RNA Virus Surveillance

**DOI:** 10.1264/jsme2.ME15171

**Published:** 2016-02-13

**Authors:** Syun-ichi Urayama, Yoshihiro Takaki, Takuro Nunoura

**Affiliations:** 1 Research and Development Center for Marine Biosciences, Japan Agency for Marine-Earth Science and Technology (JAMSTEC)2–15 Natsushima-cho, Yokosuka, Kanagawa 237–0061Japan; 2 Department of Subsurface Geobiological Analysis and Research, JAMSTEC2–15 Natsushima-cho, Yokosuka, Kanagawa 237–0061Japan

**Keywords:** RNA virus, viral metagenome, dsRNA

## Abstract

Knowledge of the distribution and diversity of RNA viruses is still limited in spite of their possible environmental and epidemiological impacts because RNA virus-specific metagenomic methods have not yet been developed. We herein constructed an effective metagenomic method for RNA viruses by targeting long double-stranded (ds)RNA in cellular organisms, which is a hallmark of infection, or the replication of dsRNA and single-stranded (ss)RNA viruses, except for retroviruses. This novel dsRNA targeting metagenomic method is characterized by an extremely high recovery rate of viral RNA sequences, the retrieval of terminal sequences, and uniform read coverage, which has not previously been reported in other metagenomic methods targeting RNA viruses. This method revealed a previously unidentified viral RNA diversity of more than 20 complete RNA viral genomes including dsRNA and ssRNA viruses associated with an environmental diatom colony. Our approach will be a powerful tool for cataloging RNA viruses associated with organisms of interest.

Viruses are the universal genetic elements associated with all three domains of life (22), and virus-host interactions impact on the status of life and surrounding ecosystems (41). Historically, viruses are most often recognized as pathogens (38), and, thus, have been studied in the field of medical and crop science. Recent advances in high-throughput sequencing technologies have enabled us to identify not only viruses associated with diseases, but also those present in natural environments including oceans (41) and soil (12). Although these sequencing technologies have opened a new era in virus identification (24), a limited number of methods have been established for virus enrichment and library construction. The diversity and distribution of viruses in non-viral nucleic acid-dominant environments, such as the intracellular environments in which viruses actually replicate, still remain unclear due to technical difficulties (16). The development of a new procedure for effective virus enrichment and library construction is required in order to understand the full spectrum of diverse viruses.

RNA sequencing (RNA-seq) is a popular method in RNA virus metagenomics and is widely used for RNA virus identification (35). Purification and library construction methods have been established for RNA viruses at the extracellular stage (7, 10, 40). However, the viral read ratio of intracellular RNA viruses (RNA viruses at the intracellular stage) in the RNA-seq library is typically < 1% because mRNA and rRNA are dominant in the total RNA fraction extracted from biological samples (25). Therefore, the enrichment of viral RNA is essential for maximizing sensitivity in the identification of novel viruses. The physical enrichment of viral particles and nuclease digestion of non-viral nucleotides has been employed to increase the viral read ratio; however, a relatively low abundance of viral reads is still observed in most studies (39). These techniques are only applicable to specific RNA viruses because not all RNA viruses form viral particles (21). In addition, difficulties are associated with capturing terminal RNA sequences in an efficient and effective manner (32) and obtaining uniform coverage using the RNA-seq method. Sample preparation methods for effective viral RNA-seq are still inadequate and the sequence information generated is biased and incomplete.

In an attempt to resolve these issues, an environmental viral metagenomic approach targeting intracellular long double-stranded RNA (dsRNA) has recently been examined (2, 6, 9, 37). Intracellular dsRNA consists of the genomes of dsRNA viruses and replicative intermediates of single-stranded RNA (ssRNA) viruses, and, thus, long dsRNA is known as an RNA virus-specific molecule and molecular marker for RNA virus infection and replication (28). Therefore, a metagenomic analysis targeting intracellular long dsRNA theoretically retrieves dsRNA and ssRNA viruses, except for ssRNA retroviruses, which do not form dsRNA in the replicative stage. In addition, it is possible to eliminate non-viral nucleic acids such as mRNA and rRNA, which dominate RNA-seq reads, by DNase I, S1 nuclease, RNase, or column chromatography (44). However, previous studies have reported technical issues with the purification of dsRNA and library construction. Random priming for the reverse transcription of dsRNA does not enable the terminal sequences of the dsRNA molecule to be determined or eliminate significant contamination by non-viral sequences. The heterogeneous sequencing depth in certain viral genome segments is also an issue associated with this method (2, 6, 9, 37). Although the full-length cDNAs of dsRNA viruses may be obtained using loop primers that are ligated to the dsRNA terminal ends for reverse transcription (8), this method is only useful for short dsRNA viruses. Therefore, to the best of our knowledge, this method has not yet been applied to a viral metagenomic analysis.

We herein established a novel strategy to obtain full-length RNA virus sequences with extremely high efficiency by applying a short dsRNA full-length cloning method (8) for physically fragmented dsRNAs. The improved method, named FLDS (fragmented and loop primer ligated dsRNA sequencing), was applied to a diatom colony in a tide pool and revealed previously unidentified RNA viruses. Our results indicate that the diversity of environmental RNA viruses has been underestimated due to the technical limitations in identifying entire RNA viromes in cellular organisms, and this technique will be a powerful tool for cataloging RNA viruses associated with organisms of interest.

## Materials and Methods

### Model and environmental samples

Mycelial plugs of *Magnaporthe oryzae* strain S-0412-II 1a, naturally infected with Magnaporthe oryzae chrysovirus 1 strain A (MoCV1-A) (45) were incubated in 0.5% yeast extract and 2% glucose liquid broth (YG broth) with reciprocal shaking (60 rpm) at 25°C for 2 weeks in the laboratory of Prof. Teraoka (Tokyo University of Agriculture and Technology). Colonies of a diatom on tidal rocks in Tokyo Bay (35.3405° N, 139.6396° E) were sampled in April 2014. After washing with distilled water, the colonies were stored at −80°C.

### Purification and fragmentation of dsRNA

DsRNA was purified as described by Okada *et al.* with a few modifications (31, 46). Briefly, the microbial sample was disrupted in liquid nitrogen in a mortar and total nucleic acids were manually extracted. DsRNA was purified twice through a micro-spin column (empty Bio-spin column; Bio-Rad Laboratories, Inc., Hercules, CA, USA) containing cellulose powder (Cellulose D; ADVANTEC, Tokyo, Japan) to obtain pure dsRNA. The dsRNA eluted from cellulose powder in MQ water was treated with DNaseI (amplification grade, Invitrogen, Carlsbad, CA, USA) and S1 nuclease (Invitrogen) in nuclease buffer (57 mM CH_3_COONa, 9.5 mM MgCl_2_, 1.9 mM ZnSO_4_, and 189 mM NaCl) and was then incubated at 37°C for 2 h. The final concentrations of CH_3_COONa, MgCl_2_, ZnSO_4_, and NaCl were adjusted to 90 mM, 15 mM, 3 mM, and 300 mM, respectively. DsRNA was purified using an RNeasy Mini Kit (Qiagen, Valencia, CA). A one-tenth volume of 10 × ShortCut buffer and 10 × MnCl provided with ShortCut RNase III (NEB Japan, Tokyo, Japan) was added to the dsRNA solution and fragmented by ultrasound at 4°C in Snap-Cap microTUBEs using a Covaris S220 (Woburn, MA, USA). The fragmentation conditions were as follows; run time 35 s, peak power 140.0 W, duty factor 2.0%, and 200 cycles/burst. Fragmented dsRNA was divided into two equal volumes, and maintained at 37°C with or without ShortCut RNase III (NEB). DsRNAs were then purified using a ZymoClean Gel RNA Recovery Kit (ZymoResearch, Orange, CA). Note that dsRNA purification from *M. oryzae* was carried out in the laboratory of Prof. Teraoka.

### cDNA synthesis and amplification for dsRNA

The PC3-T7 loop primer (5′-p-GGA TCC CGG GAA TTC GGT AAT ACG ACT CAC TAT ATT TTT ATA GTG AGT CGT ATT A-OH-3′) was ligated to fragmented dsRNA as described by Potgieter *et al.* (34), and dsRNA was then purified using the MinElute Gel Extraction Kit (Qiagen). After the addition of DMSO at a final concentration of 15% (v/v), dsRNA was denatured at 95°C for 3 min and snap-frozen in ice-water slurry. RNA was reverse transcribed into cDNA from the ligated loop primer region using the Superscript III First-Strand Synthesis System (Invitrogen). After excess and hybrid RNAs were removed (34), cDNA was desalted and concentrated using the MinElute PCR cleanup kit (Qiagen). Primary cDNA strands were re-annealed by lowering the temperature from 95 to 50°C, as described previously (30). Second strand DNA polymerization was performed using KOD-plus Neo (Toyobo, Osaka, Japan) with a primer complementary to the partial sequence of the PC3-T7 loop primer, PC2 (5′-CCG AAT TCC CGG GAT CC-3′) (34). After heat activation of KOD-plus Neo in the reaction mixture provided at 96°C for 2 min, template cDNA was added and incubated at 68°C for 5 min. After the reaction, cDNA was amplified under the following conditions: 96°C for 2 min, 25 (for MoCV1-A) or 18 (for diatom colony) cycles of 98°C for 10 s, and 68°C for 2 min. Small cDNA and primer dimers were removed using the 1.25 × SPRIselect reagent kit (Beckman Coulter, Brea, CA, USA) according to the Left Side Size Selection procedure in the manufacturer’s protocol.

### Total RNA extraction, cDNA synthesis, and library construction from an environmental sample

Total RNA was isolated from a diatom colony using the TRIzol Plus RNA Purification Kit (Invitrogen) according to the manufacturer’s protocol. The RNA fraction was treated with DNase I (Takara, Otsu, Japan). Double-stranded cDNA was synthesized from 2 μg of total RNA with random primers (9-mers) using a PrimeScript Double Strand cDNA Synthesis Kit (Takara). The resultant cDNA was quantified using a Qubit dsDNA HS Kit.

### Illumina sequencing

Ultrasound was used to fragment cDNA in Snap-Cap microTUBEs at 4°C using a Covaris S220 (Woburn, MA, USA). The fragmentation conditions were as follows; run time 55 s, peak power 175.0 W, duty factor 5.0% and 200 cycles/burst. The Illumina library was constructed with KAPA Hyper Prep Kit Illumina platforms (Kapa Biosystems, Woburn, MA, USA). The quantity of the library was evaluated using the KAPA library quantification kit (Kapa Biosystems). Each 300 bp of the paired-end sequences of each fragment were determined with the Illumina MiSeq platform (San Diego, CA, USA).

### Data assembly and processing

Raw sequence reads were processed with the CLC Genomics Workbench (CLC Bio, Aarhus, Denmark). Adaptor and primer sequences were trimmed, and low quality sequence regions were removed with default parameters. PhiX sequences derived from control libraries and experimentally contaminated sequences (< 0.05% of total reads) were also removed using a mapping tool. The consensus sequences of viral contigs were obtained *de novo* exclusively with the CLC Genomics Workbench (CLC Bio), and assemblies were manually examined and extended using the Tablet viewer (27). Using the mapping tool, each contig was confirmed to be constructed with at least 3 × sequence coverage, 10 × average coverage, and 1,000 bp in length. In cases of dominant reads (more than 10 reads) that stopped in the same position around the ends of contigs, the position was recognized as a terminal end. The predicted terminal ends of the viral genome segments were also confirmed by the presence of adjacent PCR primer sequences next to the predicted terminal sequence, except for cases of contigs with a poly(A) tail. Contigs with 70–90% nucleotide identity with other contigs were classified as the genome types of the same species. Contigs with > 90% nucleotide identity were assigned as the same genome type and only major contigs were used in further analyses. Sequences were compared against the NCBI non-redundant nucleotide and amino acid (aa) databases using BLASTN-plus and BLASTX-plus, respectively (5), and then classified by MEGAN 5.7.1. (18). A sequence analysis was performed using Genetyx-MAC software version 17.0.0 (Genetyx Corp., Tokyo, Japan) and Genetyx software version 9.1.0 (Genetyx). Most full-length small subunit rRNA sequences in the diatom colony were reconstructed from RNA-seq reads with EMIRGE (26).

### Phylogenetic analysis

Multiple alignments based on the deduced aa sequences of putative RNA-dependent RNA polymerase (RdRp) genes in dsRNA contigs were obtained using ClustalX 2.0 (23) and MEGA5 software (42). Phylogenetic analyses were conducted using MrBayes 3.2.3 (36) with the model of aa substitution, RtREV+I+G+F, selected by ProtTest2.4 (1), as judged by the Akaike information criterion (33). Bayesian analyses with the covarion parameter were run with one run and four chains for 1,000,000 generations.

### Data accession

The data sets supporting the results of this study are available in the GenBank database repository (accession nos. DDBJ: AP014890–AP014920) and Short Read Archive database (accession no. DDBJ: DRA003723 and DRA003724).

## Results

### Application of FLDS to a segmented dsRNA virus

**Fig. 1:**
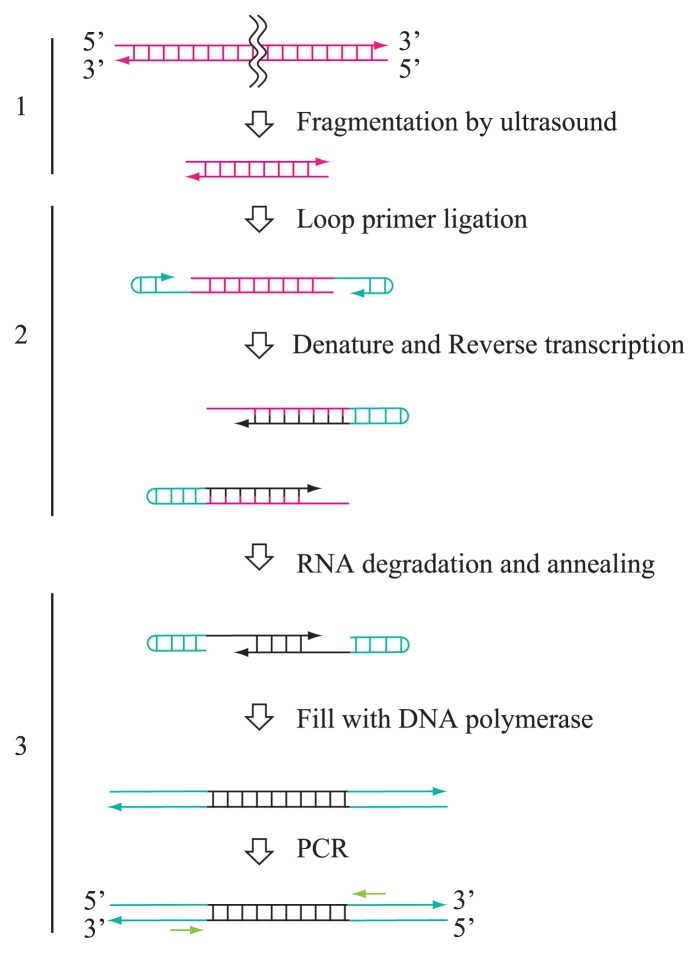
Schematic work flow of FLDS. 1. Fragmentation of dsRNA by ultrasound. 2. Ligation of a loop primer on 3′-terminal ends and reverse transcription. 3. Selective duplex formation of cDNA from dsRNA, and PCR amplification. Details of the FLDS method are described in the Materials and Methods section.

The novel dsRNA purification and library construction method, named FLDS, consists of cellulose column chromatography, the physical fragmentation of dsRNA, cDNA synthesis using a loop primer, and the PCR amplification of cDNA (Fig. 1). The purification of dsRNA was achieved by the repeated affinity purification of dsRNA using cellulose powder and the enzymatic removal of ssRNA and DNA. Purified dsRNAs were fragmented using ultrasound to retrieve all types of dsRNA viruses in order to apply the previously reported full-length dsRNA cloning method using a loop primer (8). The full-length dsRNA cloning method requires overlapped cDNAs synthesized from both terminal ends for further cDNA amplification, and was only applicable to short dsRNA molecules. Reverse transcription was initiated from the ligated loop primer on both ends of the dsRNA fragment. cDNA was then thermally denatured to allow annealing of single-stranded cDNA with the complementary sequence in the 3′ terminal region. The single-stranded regions of annealed cDNA were filled in with DNA polymerase. The double-stranded cDNA derived from dsRNA was amplified by PCR with a single primer (PC2) in order to obtain sufficient cDNA to construct a sequencing library.

Mycelial MoCV1-A was used to test the feasibility of this method. Since PCR amplicons were not observed in the dsRNA-specific RNaseIII-treated sample prior to reverse transcription, most of the amplicons (cDNA) were likely to have been derived from dsRNA (Fig. S1). The results of the sequencing analysis indicated that 99.1% of total reads were derived from the MoCV1-A genome (Table S1). Five contigs obtained by *de novo* assembly were identical to the entire region of the MoCV1-A genome segments attained using a conventional cloning and sequencing method (44, 45) with > 99.9% identity (Table S2). Read mapping on MoCV1-A genomes (Fig. S2) showed that the sequence coverage of terminal regions was generally higher than that of the central regions of each segment with few exceptions. No obvious relationship was observed between read coverage and GC content (Fig. S2). These results indicated that FLDS effectively enriched dsRNA reads, thereby allowing the retrieval of complete genome sequences including terminal regions without the requirement for the additional rapid amplification of cDNA ends (RACE).

### FLDS analysis in an environmental diatom colony

**Fig. 2:**
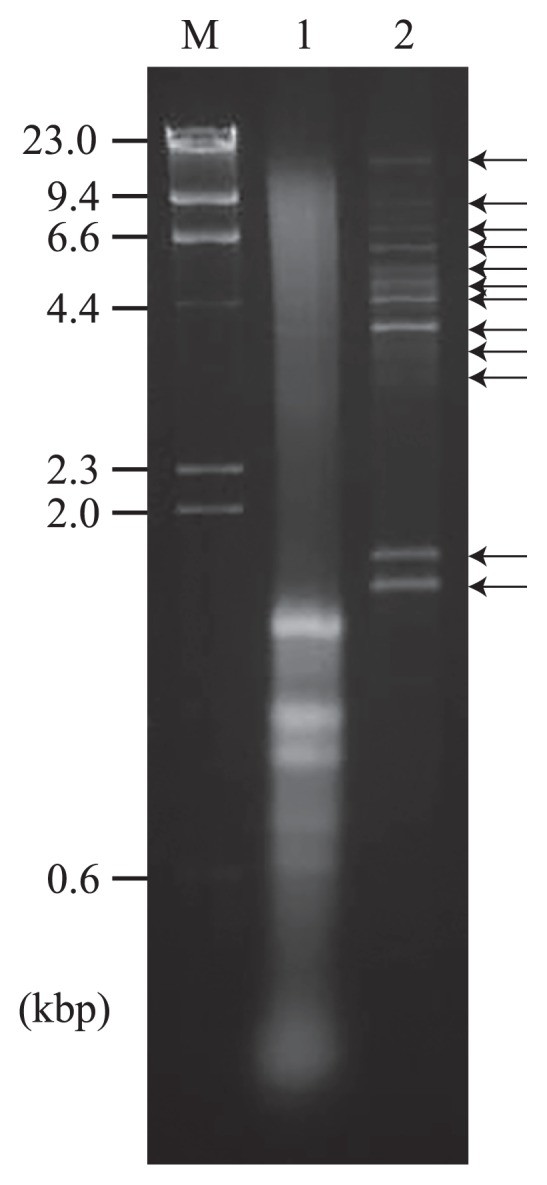
Agarose gel electrophoresis of purified nucleic acids from a diatom colony. Nucleic acids were stained with ethidium bromide. Lane M, 300 ng of HindIII-digested λ DNA; lane 1, total nucleic acids extracted from 5 mg (wet weight) of the diatom colony; lane 2, purified dsRNA extracted from 1 g (wet weight) of the diatom colony.

Gel electrophoresis showed that the total long dsRNA fraction from the diatom colony contained at least ten dsRNA segments, whereas genomic DNA and rRNA were the predominant in total nucleic acids (Fig. 2). Total dsRNA extracted from 1 g of the diatom colony was analyzed using the FLDS method. PCR amplicons were not observed in the dsRNA-specific RNaseIII-treated sample prior to reverse transcription (Fig. S3). As a result of *de novo* assembly and manual extension, we obtained 42 composite viral contigs (Table 1 and Table S3). More than 98.2% of reads were mapped to these 42 contigs (Table 2) as in the case of the model experiment described above. Both terminal ends of 31 of the viral contigs were identified and recognized as full-length viral genome segments. The terminal sequences of the full-length segments were used to identify segment compositions for some of the viral species because terminal sequences are highly conserved between segments in some dsRNA viral genomes for viral RNA replication and/or encapsidation (19).

Based on aa sequence similarities (E-value ≥ 1 × 10^−5^) in the predicted protein-encoding sequences (CDSs), the number of genome segments in related viruses, and terminal conserved sequences in each segment of a single virus, we identified 22 viral putative composite genomes out of 31 full-length viral segments. Sequence similarities between the 22 putative viral composite genomes were used to classify them into 19 putative viral species, and each of the two genome types was identified in three species (Table 1). Seventeen dsRNA and two ssRNA viral species were identified and named Diatom Colony-Associated dsRNA virus 1–17 (DCADSRV-1–17) and Diatom Colony-Associated ssRNA virus 1–2 (DCASSRV-1–2) (Table 1). Since ssRNA viruses form an RNA duplex as an intermediate in genome replication, these contigs were most likely derived from replicating ssRNA viruses (11) and not from contaminant ssRNA. An additional seven full-length viral segments with predicted CDSs were also identified; however, we were unable to determine the combination of their segments or reconstruct viral genomes based on information from previously reported viruses. Thus, these viral segments were assigned as Diatom Colony-Associated Virus-Like RNA Segments (DCAVLRS-1–7).

### Comparison between FLDS and total RNA-seq

Total RNA from the diatom colony was also investigated using shotgun RNA-seq in order to determine the active organisms of the colony and the abundance of viral RNA genomes in total RNA. Sequence reads derived from rRNA were identified using EMIRGE (26). The results of the analysis revealed that 56% of all trimmed reads were rRNA sequences, while 37.2% of all reads showed more than 99% identity to 18S and 23S rRNA from the diatom *Achnanthes brevipes*. In addition, 4.1 and 6.2% of reads belonged to the other diatom genus *Cylindrotheca* and chlorophyte genus *Cladophora*, respectively. The relative abundance of the rRNA reads was shown in Table S4.

**Fig. 3:**
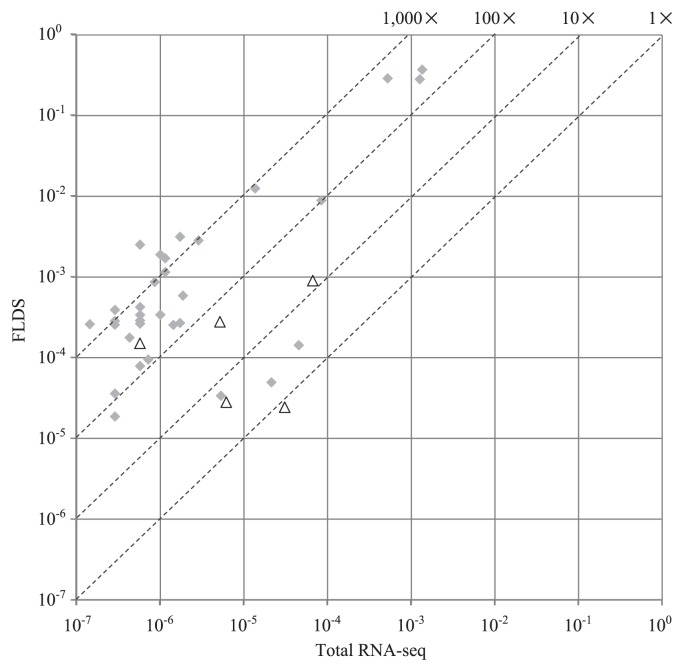
Comparison of mapped read frequencies for each viral contig between FLDS and total RNA-seq. Plots indicate each viral contig. The rhombus and triangle plots show dsRNA and ssRNA viral contigs, respectively. 10^0^–10^−7^ represent the frequencies of reads in each library. Dotted lines with 1×, 10×, 100×, or 1000× show a higher viral read frequency than that with an RNA-seq analysis. Reads mapped with nine contigs found in FLDS were not found in total RNA-seq.

**Fig. 4:**
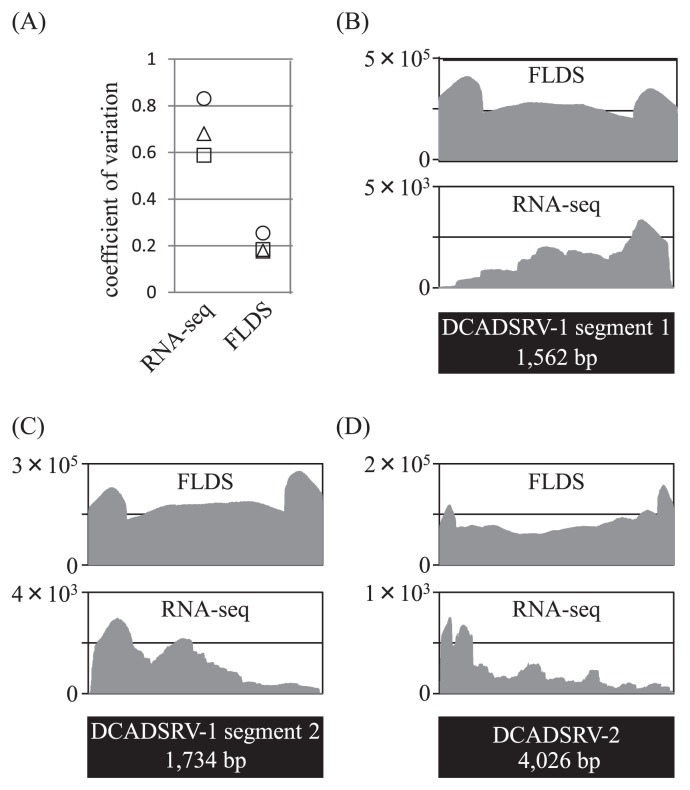
Comparison of coverage uniformity between FLDS and RNA-seq. DsRNA segments with an average depth of > 200 in RNA-seq were used for the analysis. (A) Coefficient of variation (the ratio of the standard deviation to the mean coverage). Values were plotted on viral dsRNA segments of DCADSRV-1 segment 1 (square), DCADSRV-1 segment 2 (triangle), and DCADSRV-2 (circle), and were plotted on the Y axis. (B–D) Genomic coverage of each viral segment from the FLDS (upper graph) and RNA-seq (lower graph) analysis.

Only 0.3% of reads from total RNA-seq was mapped on the major viral contigs obtained using FLDS with a read mapping algorithm in the CLC workbench (Table 2). Comparisons of the relative read frequencies of each major viral contig between total RNA-seq and FLDS revealed that FLDS achieved 0.8–4372.3-fold enrichment for each viral contig (653.2 mean) (Fig. 3). FLDS also had apparent advantages in uniform read coverage and efficiency for retrieving terminal sequences (Fig. 4). Sequence reads for ssRNA viruses in FLDS were also more abundant than when RNA-seq was used for four out of five ssRNA contigs. In addition, by *de novo* assembly, only six partial viral contigs were obtained using RNA-seq, and no viral contigs specific for total RNA-seq were found. Accordingly, we concluded that FLDS is more efficient than total RNA-seq for the detection and identification of RNA viruses, with the exception of retroviruses, which theoretically cannot be identified using FLDS.

### Phylogenetic analysis and characterization of viral RNA genomes

A phylogenetic analysis of viral RNA replicases (RNA-dependent RNA polymerase; RdRp) presented the phylogenetic relationship between viral genomes from a diatom colony and known RNA viruses (Fig. S4). Viruses belonging to the family *Totiviridae* harbor non-segmented dsRNA genomes and form isometric virions that infect either fungi or protozoa (21). Thirteen composite genomes of *Totiviridae-*related viruses were identified and classified into four clades distinct from the five characterized genera of *Totiviridae* (clades a–d in Fig. S4A). Clade c was the sister clade of the proposed genus “*Trichomonasvirus*” and clade d included *Ustilaginoidea virens* RNA virus 1 (UvRV1). In general, −1 ribosomal frameshift signals [the XXXYYYZ motif (4), in which XXX may be any three identical nucleotides, YYY may be either AAA or UUU, and Z may be A, U, or C] or +1 ribosomal frameshift signals [CCCUUUU (14) or UCCUUUCGU (47)] were located in the upstream region of the 2^nd^ CDS, and were used in the expression of overlapping viral genes such as the *pol* (RdRp) of *Totivirus* and *Leishmaniavirus*. These regions were examined in an attempt to better classify the identified viruses. However, as in the case of UvRV1, −1 or +1 ribosomal frameshift signals were not found in any of the *Totiviridae* genomes obtained in this study. CDSs in the predicted *Totiviridae* virus-like segments DCAVLRS-3 and DCAVLRS-4 showed significant similarities with the *gag* (coat protein; CP) and *pol* (RdRp) of known totiviruses, respectively. *Totiviridae* genomes consist of a single genome segment that encodes the two essential CDSs, whereas DCAVLRS-3 and -4 lacked *pol* and *gag*, respectively. These two segments harbored nine identical 5′-terminal nucleotide sequences, which were distinguishable from the other identified terminal viral sequences. Genomic features implied that DCAVLRS-3 and -4 may be parts of a bisegmented viral genome. RdRp in DCADSRV-1 segment 2 showed significant homology with that in fox *Picobirnavirus*, a member of the *Picobirnaviridae*, although DCADSRV-1 was phylogenetically distinct from the known *Picobirnaviridae* viruses (Fig. S4B). Picobirnaviruses are small, non-enveloped, bisegmented dsRNA viruses that infect animals and humans (21). The genome structure of DCADSRV-1 was similar to that of the known *Picobirnaviridae* (21). DCADSRV-14 was classified into the genus *Deltapartitivirus* of the family *Partitiviridae* based on the predicted RdRp sequence (Fig. S4C) (29). To date, all of the alphacryptoviruses have been identified from plants including the angiosperm, gymnosperm, and chlorophytes (29). rRNA sequences belonging to the *Streptophyta*, including land plants, have not yet been detected by an RNA-seq analysis, whereas *Cladophora* sp. of the *Chlorophyta*, a sister division of *Streptophyta*, were detected (Table S4). The CDSs of DCADSRV-15 and a few viral contigs showed significant homology with viruses belonging to the *Endornaviridae* (dsRNA), *Naranviridae* (ssRNA), or *Hypoviridae* (ssRNA), whose virion formation has not yet been observed.

In the ssRNA viral population, RdRp in DCASSRV-1 presented a close relationship with Border disease virus—BD31 (E-value = 4 × 10^−15^), a member of the genus *Pestivirus* of the family *Flaviviridae*, which consists of the arthropodborne pathogens of humans and other animals. The genome size and CDS structure of DCASSRV-1 (11.4 kb) were similar to those of *Flaviviridae* (9.6–12.3 kb) (21), and the phylogenetic tree of RdRp indicated that DCASSRV-1 was not classified into the three known *Flaviviridae* genera (Fig. S4D). A phylogenetic analysis of RdRp in DCASSRV-2 suggested that the RNA virus was classified into the genus *Mitovirus*, which has a non-segmented ssRNA genome, infects the mitochondria of fungi, and lacks viral particles (Fig. S4E). The presence of multiple UGA codons suggested that the putative coding strand of DCASSRV-2 was likely to be translated in mitochondria. The genome size of DCASSRV-2 (4.5 kb) was larger than those of the known mitoviruses (2.3–3.6 kb) (17).

## Discussion

This study revealed the presence of novel RNA viruses associated with a diatom colony and inferred the unexpected evolutionary relationship between environmental viruses and pathogenic animal viruses. Among the RNA viral genomes obtained in this study, some dominant populations showed greater similarities to fungal viruses than to known diatom viruses; however, several ssDNA and ssRNA viruses have already been identified from marine diatoms (20, 43). We cannot exclude the possibility that these viral genomes were derived from fungi associated with a diatom colony, but it is more likely that they came from the major components of a diatom colony because of their high abundance in the RNA viral metagenomic library. Since extracellular viral particles have been a major target of virus surveillance and isolation, information on intracellular viruses in microorganisms is very limited (13, 37). Therefore, the accumulation of knowledge on intracellular RNA viruses infecting diverse host organisms is essential for understanding the evolution and distribution of RNA viruses.

FLDS revealed 22 full-length and some partial composite viral RNA genomes associated with a diatom colony by *de novo* assembly. These were classified into five dsRNA (*Totiviridae*, *Endornaviridae*, *Picobirnaviridae*, *Cystoviridae*, and *Partitiviridae*) and four ssRNA (*Flaviviridae*, *Narnaviridae*, *Virgaviridae*, and *Hypoviridae*) virus families. To the best of our knowledge, this is the largest number of full-length genome sequences of novel RNA viruses identified in one metagenomic library. The viral RNA community successfully detected in this study consisted of dsRNA viruses with or without virion formation and ssRNA viruses detected as replicative intermediates. Our results suggest that FLDS has the potential to detect a wide range of RNA viruses, excluding retroviruses.

Several studies have been performed using metagenomic analyses targeting dsRNA with Next-Generation Sequencing technology. In these studies, viral read abundance reached a maximum of 52.7% (2). In contrast, FLDS provided extremely high viral read abundance. The improvement in viral read rates with FLDS was likely derived from [1] a combination of repeating cellulose powder column chromatography and subsequent enzymatic treatment, [2] the fragmentation and efficient thermal denaturation of dsRNA prior to cDNA synthesis, and [3] the selective duplex formation of dsRNA-derived cDNA prior to PCR amplification. Furthermore, FLDS also presented advantages in reconstructing complete genome sequences including terminal regions, which are difficult to obtain using RNA-seq and random priming methods (3). The complete sequences of viral RNA segments are beneficial for the identification of RNA virus segments, particularly in cases in which coding CDSs did not show significant similarities with viral CDSs in databases. The application of a fulllength dsRNA cloning method using a PC3-T7 loop primer (8) to fragmented dsRNA enabled us to determine the terminal regions of long dsRNA genomes. Since T4 RNA ligase requires a 5′ phosphoryl-terminated nucleic acid donor (PC3-T7 loop primer) and 3′ hydroxyl-terminated nucleic acid acceptor for ligation activity, dsRNA fragments with 3′ terminal phosphate were not used as substrates. The terminal structures of dsRNA fragmented by ultrasound have not been reported. However, in the case of dsDNA fragmented by ultrasound, double-strand breaks occur preferentially in 5′-CpG-3′ dinucleotides, and the phosphate group is at the 5′ side of G in the products (15). In this study, fragmented dsRNAs were successfully converted into cDNA and amplified. Taking this into consideration, dsRNA fragmentation using ultrasound with Covaris S220 also produced 3′ hydroxyl-terminated fragments. Furthermore, the lack of any modifications to the 3′ hydroxyl-terminal of viral RNA genomes (21) also allowed us to retrieve the terminal regions of the RNA viral genome.

Total RNA-seq is considered to be a less-biased method for identifying RNA viruses despite the very low abundance of viral reads in general. In the present study, FLDS enriched the viral RNA reads by > 300-fold that with total RNA-seq (Table 2). Notably, FLDS produced significantly more ssRNA viral reads than total RNA-seq; however, FLDS only has the ability to detect ssRNA viruses at the replicative stage. Moreover, FLDS showed more uniform read coverage than RNA-seq. These results indicate that FLDS is more effective than total RNA-seq for revealing all RNA viruses in cellular organisms.

## Supplementary Material



## Figures and Tables

**Table 1:** List of complete composite genomes of RNA viruses and full-length virus-like RNAs obtained from a diatom colony obtained using FLDS.

RNA virus species	Accession	Description	Size (nt)	Num. of mapped reads	Average coverage	BlastX analysis		

						Top Hit for each CDS, Virus family	E-value	Protein
DCADSRV-1a)	AP014890	segment 1	1,734	1,301,278	191,942	—	—	—
	AP014891	segment 2	1,562	1,717,396	279,580	Fox *Picobirnavirus* *Picobirnaviridae*	1 × 10^−33^	RdRp

DCADSRV-2	AP014892		4,026	1,337,570	83,876	*Ustilaginoidea virens* nonsegmented virus1 Not assigned	5 × 10^−15^	RdRp

DCADSRV-3	AP014893		4,911	14,544	703	*Ustilaginoidea virens* RNA virus1 *Totiviridae*	2 × 10^−63^	RdRp

DCADSRV-4	AP014894	Genome type A	4,982	12,325	591	*Aspergillus mycovirus* 178 *Totiviridae*	4 × 10^−69^	RdRp
DCADSRV-4	AP014895	Genome type B	4,979	1,074	52	*Ustilaginoidea virens* RNA virus1 *Totiviridae*	5 × 10^−69^	RdRp

DCADSRV-5	AP014896		5,252	7,863	359	*Aspergillus foetidus* slow virus 1 *Totiviridae*	3 × 10^−74^	RdRp

DCADSRV-6	AP014897		4,939	2,720	131	*Aspergillus mycovirus* 178 *Totiviridae*	2 × 10^−66^	RdRp

DCADSRV-7	AP014898		5,327	1,957	87	*Gremmeniella abietina* RNA virus L1 *Totiviridae*	3 × 10^−123^	RdRp
						*Ustilaginoidea virens* RNA virus 3 *Totiviridae*	2 × 10^−56^	CP

DCADSRV-8	AP014899		4,660	1,163	60	*Aspergillus foetidus* slow virus 1 *Totiviridae*	8 × 10^−57^	RdRp

DCADSRV-9	AP014900	Genome type A	4,844	1,198	60	*Magnaporthe oryzae* virus 2 *Totiviridae*	1 × 10^−65^	RdRp
DCADSRV-9	AP014901	Genome type B	4,845	364	18	*Aspergillus foetidus* slow virus 1 *Totiviridae*	2 × 10^−66^	RdRp

DCADSRV-10	AP014902		5,082	1,244	59	*Rosellinia necatrix* victorivirus 1 *Totiviridae*	2 × 10^−108^	RdRp
						*Ustilaginoidea virens* RNA virus 1 *Totiviridae*	6 × 10^−50^	CP

DCADSRV-11	AP014903		5,160	1,173	55	*Ustilaginoidea virens* RNA virus1 *Totiviridae*	4 × 10^−128^	RdRp
						*Ustilaginoidea virens* RNA virus 1 *Totiviridae*	8 × 10^−64^	CP

DCADSRV-12	AP014904		5,941	1,219	49	*Beauveria bassiana* RNA virus 1 *Totiviridae*	1 × 10^−40^	RdRp

DCADSRV-13	AP014905		4,671	820	42	*Aspergillus foetidus* slow virus 1 *Totiviridae*	4 × 10^−58^	RdRp

DCADSRV-14a)	AP014906	segment 1	1,576	438	67	Persimmon cryptic virus *Partitiviridae*	3 × 10^−97^	RdRp
	AP014907	segment 2	1,490	274	43	—	—	—

DCADSRV-15	AP014908		12,172	1,482	29	*Chalara endornavirus* CeEV1 *Endornaviridae*	1 × 10^−115^	Polyprotein

DCASSRV-1	AP014912		11,413	1,011	21	Border disease virus—BD31 *Flaviviridae*	4 × 10^−15^	Polyprotein

DCASSRV-2	AP014913		4,586	4,153	224	*Tuber excavatum* mitovirus *Narnaviridae*	5 × 10^−20^	RdRp

DCADSRV-16	AP014909		6,635	8,735	310	*Rhizoctonia fumigata* mycovirus Not assigned	4 × 10^−10^	RdRp

DCADSRV-17	AP014910	Genome type A	5,907	5,325	218	dsRNA virus environmental sample Not assigned	7 × 10^−14^	RdRp
DCADSRV-17	AP014911	Genome type B	5,909	1,564	63	*Botrytis porri* RNA virus 1 Not assigned	1 × 10^−13^	RdRp

DCAVLRS-1	AP014914	Interrupted RdRp	4,567	57,802	3,039	*Ustilaginoidea virens* nonsegmented virus 1 Not assigned	3 × 10^−11^	RdRp

DCAVLRS-2	AP014915	Interrupted RdRp	4,786	41,181	2,100	*Ustilaginoidea virens* nonsegmented virus 1 Not assigned	2 × 10^−11^	RdRp

DCAVLRS-3	AP014916	CP only	3,458	13,140	876	*Ustilaginoidea virens* RNA virus 1 *Totiviridae*	2 × 10^−41^	CP

DCAVLRS-4	AP014917	RdRp only	3,190	3,995	294	*Magnaporthe oryzae* virus 2 *Totiviridae*	2 × 10^−123^	RdRp

DCAVLRS-5	AP014918	CP only	3,262	1,331	96	*Phomopsis vexans* RNA virus *Totiviridae*	5 × 10^−47^	CP

DCAVLRS-6	AP014919	RdRp only	3,325	891	65	*Ustilaginoidea virens* RNA virus 3 *Totiviridae*	6 × 10^−102^	RdRp

DCAVLRS-7	AP014920	Interrupted RdRp	1,986	164	20	*Flammulina velutipes* browning virus *Partitiviridae*	4 × 10^−63^	RdRp

a) The classification was based on the shared 5′ terminal sequences in paired segments, whereas CDSs in the segments that did not show significant similarities with genes in databases.

**Table 2:** Classification of next-generation sequencing reads obtained by FLDS and total RNA-seq.

	FLDS		total RNA-seq	
	
	Num. of reads	rate (%)	Num. of reads rate	(%)
Trimmed	4,631,738	100.0	6,979,561	100.0
Major viral reads	4,549,629	98.2	24,036	0.3
Unmapped reads (include minor viral reads)	82,109	1.7	6,955,525	99.6

## References

[1] Abascal F, Zardoya R, Posada D (2005). Prottest: Selection of best-fit models of protein evolution. Bioinformatics.

[2] Al Rwahnih M, Daubert S, Golino D, Rowhani A (2009). Deep sequencing analysis of RNAs from a grapevine showing Syrah decline symptoms reveals a multiple virus infection that includes a novel virus. Virology.

[3] Alfson KJ, Beadles MW, Griffiths A (2014). A new approach to determining whole viral genomic sequences including termini using a single deep sequencing run. J Virol Methods.

[4] Brierley I, Jenner AJ, Inglis SC (1992). Mutational analysis of the “slippery-sequence” component of a coronavirus ribosomal frame-shifting signal. J Mol Biol.

[5] Camacho C, Coulouris G, Avagyan V, Ma N, Papadopoulos J, Bealer K, Madden TL (2009). Blast+: Architecture and applications. BMC Bioinformatics.

[6] Coetzee B, Freeborough MJ, Maree HJ, Celton JM, Rees DJ, Burger JT (2010). Deep sequencing analysis of viruses infecting grapevines: Virome of a vineyard. Virology.

[7] Culley AI, Lang AS, Suttle CA (2006). Metagenomic analysis of coastal RNA virus communities. Science.

[8] Darissa O, Willingmann P, Adam G (2010). Optimized approaches for the sequence determination of double-stranded RNA templates. J Virol Methods.

[9] Decker CJ, Parker R (2014). Analysis of double-stranded RNA from microbial communities identifies double-stranded RNA virus-like elements. Cell Rep.

[10] Djikeng A, Kuzmickas R, Anderson NG, Spiro DJ (2009). Metagenomic analysis of RNA viruses in a fresh water lake. PLoS One.

[11] Dodds JA, Morris TJ, Jordan RL (1984). Plant viral double-stranded RNA. Annu Rev Phytopathol.

[12] Fierer N, Breitbart M, Nulton J (2007). Metagenomic and small-subunit rRNA analyses reveal the genetic diversity of bacteria, archaea, fungi, and viruses in soil. Appl Environ Microbiol.

[13] Ghabrial SA, Castón JR, Jiang D, Nibert ML, Suzuki N (2015). 50-plus years of fungal viruses. Virology.

[14] Goodman RP, Freret TS, Kula T (2011). Clinical isolates of *Trichomonas vaginalis* concurrently infected by strains of up to four trichomonasvirus species (family *Totiviridae*). J Virol.

[15] Grokhovsky SL (2006). Specificity of DNA cleavage by ultrasound. Mol Biol.

[16] Hall RJ, Wang J, Todd AK (2014). Evaluation of rapid and simple techniques for the enrichment of viruses prior to metagenomic virus discovery. J Virol Methods.

[17] Hillman BI, Cai G (2013). The family *Narnaviridae*: Simplest of RNA viruses. Adv Virus Res.

[18] Huson DH, Mitra S, Ruscheweyh HJ, Weber N, Schuster SC (2011). Integrative analysis of environmental sequences using MEGAN4. Genome Res.

[19] Hutchinson EC, von Kirchbach JC, Gog JR, Digard P (2010). Genome packaging in influenza A virus. J Gen Virol.

[20] Kimura K, Tomaru Y (2015). Discovery of two novel viruses expands the diversity of single-stranded DNA and single-stranded RNA viruses infecting a cosmopolitan marine diatom. Appl Environ Microbiol.

[21] King AMQ, Adams MJ, Carstens EB, Lefkowitz EJ (2012). Virus taxonomy: Classification and nomenclature of viruses: Ninth report of the international committee on taxonomy of viruses.

[22] Koonin EV (2010). The two empires and three domains of life in the postgenomic age. Nat Educ.

[23] Larkin MA, Blackshields G, Brown NP, Chenna R, McGettigan PA, McWilliam H, Valentin F, Wallace IM, Wilm A, Lopez R (2007). Clustal W and clustal X version 2.0. Bioinformatics.

[24] Lipkin WI (2013). The changing face of pathogen discovery and surveillance. Nat Rev Microbiol.

[25] Matranga CB, Andersen KG, Winnicki S (2014). Enhanced methods for unbiased deep sequencing of Lassa and Ebola RNA viruses from clinical and biological samples. Genome Biol.

[26] Miller CS, Baker BJ, Thomas BC, Singer SW, Banfield JF (2011). EMIRGE: Reconstruction of full-length ribosomal genes from microbial community short read sequencing data. Genome Biol.

[27] Milne I, Bayer M, Cardle L, Shaw P, Stephen G, Wright F, Marshall D (2010). Tablet—next generation sequence assembly visualization. Bioinformatics.

[28] Morris TJ, Dodds JA (1979). Isolation and analysis of double-stranded RNA from virus-infected plant and fungal tissue. Phytopathology.

[29] Nibert ML, Ghabrial SA, Maiss E, Lesker T, Vainio EJ, Jiang D, Suzuki N (2014). Taxonomic reorganization of family *Partitiviridae* and other recent progress in partitivirus research. Virus Res.

[30] Nomikou K, Dovas CI, Maan S, Anthony SJ, Samuel AR, Papanastassopoulou M, Maan NS, Mangana O, Mertens PP (2009). Evolution and phylogenetic analysis of full-length VP3 genes of eastern mediterranean bluetongue virus isolates. PLoS One.

[31] Okada R, Kiyota E, Moriyama H, Fukuhara T, Natsuaki T (2015). A simple and rapid method to purify viral dsRNA from plant and fungal tissue. J Gen Plant Pathol.

[32] Ozsolak F, Milos PM (2011). RNA sequencing: Advances, challenges and opportunities. Nat Rev Genet.

[33] Posada D, Buckley TR (2004). Model selection and model averaging in phylogenetics: Advantages of akaike information criterion and bayesian approaches over likelihood ratio tests. Syst Biol.

[34] Potgieter AC, Page NA, Liebenberg J, Wright IM, Landt O, van Dijk AA (2009). Improved strategies for sequence-independent amplification and sequencing of viral double-stranded RNA genomes. J Gen Virol.

[35] Pybus OG, Rambaut A (2009). Evolutionary analysis of the dynamics of viral infectious disease. Nat Rev Genet.

[36] Ronquist F, Huelsenbeck JP (2003). MrBayes 3: Bayesian phylogenetic inference under mixed models. Bioinformatics.

[37] Roossinck MJ, Saha P, Wiley GB, Quan J, White JD, Lai H, Chavarria F, Shen G, Roe BA (2010). Ecogenomics: Using massively parallel pyrosequencing to understand virus ecology. Mol Ecol.

[38] Roossinck MJ (2011). The good viruses: Viral mutualistic symbioses. Nat Rev Microbiol.

[39] Shah JD, Baller J, Zhang Y, Silverstein K, Xing Z, Cardona CJ (2014). Comparison of tissue sample processing methods for harvesting the viral metagenome and a snapshot of the RNA viral community in a turkey gut. J Virol Methods.

[40] Steward GF, Culley AI, Mueller JA, Wood-Charlson EM, Belcaid M, Poisson G (2013). Are we missing half of the viruses in the ocean?. ISME J.

[41] Suttle CA (2005). Viruses in the sea. Nature.

[42] Tamura K, Peterson D, Peterson N, Stecher G, Nei M, Kumar S (2011). MEGA5: Molecular evolutionary genetics analysis using maximum likelihood, evolutionary distance, and maximum parsimony methods. Mol Biol Evol.

[43] Tomaru Y, Toyoda K, Suzuki H, Nagumo T, Kimura K, Takao Y (2013). New single-stranded DNA virus with a unique genomic structure that infects marine diatom *Chaetoceros setoensis*. Scientific reports.

[44] Urayama S, Kato S, Suzuki Y, Aoki N, Le MT, Arie T, Teraoka T, Fukuhara T, Moriyama H (2010). Mycoviruses related to chrysovirus affect vegetative growth in the rice blast fungus *Magnaporthe oryzae*. J Gen Virol.

[45] Urayama S, Ohta T, Onozuka N, Sakoda H, Fukuhara T, Arie T, Teraoka T, Moriyama H (2012). Characterization of Magnaporthe oryzae chrysovirus 1 structural proteins and their expression in *Saccharomyces cerevisiae*. J Virol.

[46] Urayama S, Yoshida-Takashima Y, Yoshida M, Tomaru Y, Moriyama H, Takai K, Nunoura T (2015). A new fractionation and recovery method of viral genomes based on nucleic acid composition and structure using tandem column chromatography. Microbes Environ.

[47] Yewdell JW, Ince WL (2012). Virology. Frameshifting to PA-X influenza. Science.

