# Supplementary material for: FLDS: A Comprehensive dsRNA Sequencing Method for Intracellular RNA Virus Surveillance
Source: Microbes Environ. 2016 Feb 13;31(1):33–40. doi: 10.1264/jsme2.ME15171 (PMC4791113; doi:10.1264/jsme2.ME15171)
Supplement: Supplementary file 1 [file 31_33_s1.pdf]

Table S1: Origins of test FLDS reads.

Blastn	Num. of reads	Reads (%)
MoCV1-A	186,830	99.09
MoCV1-A chimera	36	0.01
Host fungal sequence	37	0.01
No hits	1,508	0.79
Others	132	0.07

Table S2: Overview of re-sequencing by FLDS for the MoCV1-A genome consisting of five segments.

MoCV1-A segment	Reference length	Assembled length	Identity (%)	Total read count	Average coverage
dsRNA1	3,554	3,554	100.0	7,979	499.2
dsRNA2	3,250	3,250	99.9	31,532	2,093.1
dsRNA3	3,074	3,074	100.0	47,813	3,398.3
dsRNA4	3,043	3,043	100.0	79,226	5,617.5
dsRNA5	2,879	2,879	99.9	20,280	1,508.4

Table S3: Viral incomplete composite genomes from a diatom colony obtained using FLDS.

RNA contig name	Size (nt)	Num. of mapped reads	Average coverage	BlastX analysis		
				Top Hit, Virus family	E-value	Protein
Contig-1	5,005	658	29	*Armigeres subalbatus* virus SaX06-AK20 *Totiviridae*	6×10^{-28}	CP
Contig-2	7,822	697	22	Potato mop-top virus *Virgaviridae*	4×10^{-24}	RdRp
Contig-3	6,812	290	10	*Pseudomonas phage phi12* *Cystoviridae*	0.011[a)]	RdRp
Contig-4	1,690	309	31	*Cryphonectria hypovirus 1* *Hypoviridae*	7×10^{-16}	hypothetical protein
Contig-5	2,837	228	18	Penaeid shrimp infectious myonecrosis virus *Totiviridae*	4×10^{-54}	RdRp
Contig-6	12,744	1,398	27	Grapevine endophyte endornavirus *Endornaviridae*	1×10^{-136}	Polyprotein
Contig-7	2,499	130	13	Rhizoctonia mitovirus 1 RS002 *Narnaviridae*	8×10^{-40}	RdRp
Contig-8	3,513	183	12	*Beauveria bassiana* victorivirus NZL/1980 *Totiviridae*	1×10^{-13}	CP
Contig-9	3,176	166	12	*Coniothyrium minitans* RNA virus *Totiviridae*	8×10^{-110}	RdRp
Contig-10	2,476	112	10	*Phytophthora infestans* RNA virus 4 *Narnaviridae*	1×10^{-8}	RdRp
Contig-11	1,013	64	14	*Helicobasidium mompa totivirus 1-17* *Totiviridae*	3×10^{-42}	RdRp

[a)] The predicted amino acid sequence of Contig-3 showed significant homology ($2e^{-16}$) with previously identified, but unpublished Cystovirus.

Table S4: Relative abundance of the sequence reads mapped on rRNA sequences constructed by EMIRGE at the phylum level with more than 0.1% relative abundance

Domain	Phylum<br>Species	Ratio (%)
Eucaryota	Stramenopiles[a)]	80.1
	Achnanthes sp.	65.8
	Cylindrotheca sp.	6.7
	others	7.4
	Viridiplantae	10.7
	Cladophora sp.	10.5
	others	0.1
	Opisthokonta	1.3
	Amoebozoa	0.5
	Alveolata	0.4
Bacteria	Bacteroidetes/Chlorobi group	1.0
	Proteobacteria	0.5
	Cyanobacteria	0.2
Others	Other cellular organisms	2.3
	Not assigned	2.3

a) Reference sequences were classified using MEGAN software.

Table S5: Protein accession numbers for the phylogenetic analysis of RNA viruses.

Virus	abbreviations	Accession number
Aspergillus foetidus slow virus 1	AfV-S1	CCD33024.1
Aspergillus mycovirus 178	AsV178	ABX79995.1
Atkinsonella hypoxylon virus	AhV-2H	AAA61829.1
Beauveria bassiana RNA virus 1	BbRV1	CCC42235.1
Beet cryptic virus 1	BCV-1-Hun	YP_002308574.1
Beet cryptic virus 3	BCV3	AAB27624.1
Bell pepper endornavirus	BPEV	BAK52155.1
Black raspberry cryptic virus	BrCV	ABU55400.1
Botryotinia fuckeliana partitivirus 1	BfPV1	YP_001686789.1
Cell fusing agent virus	CFAV	NP_041725.1
Chalara endornavirus CeEV1	CeEV1	ADN43901.1
Coniothyrium minitans RNA virus	CmRV	AAO14999.1
Cryphonectria parasitica mitovirus 1-NB631	CMV-1	AAA61703.1
Cryptosporidium parvum virus 1	CSpV1-KSU1	AAC47805.1
Discula destructiva virus 1	DdV1-247	NP_116716.1
Dromedary picobirnavirus	DroPBV	KM573805.1
Enterobacteria phage MS2	MS2	ACY07217.1
Epichloe festucae virus 1	EfV-1	CAK02788.1
Feline picobirnavirus	FelPBV	KF792838.1
Fox picobirnavirus	FoxPBV-F5	AGK45545.1
Grapevine endophyte endornavirus	GeEV	YP_007003829.1
Gremmeniella abietina RNA virus L1	GaRV-L1	AAK11656.1
Gremmeniella abietina RNA virus MS1	GaRV-MS1	NP_659027.1
Gremmeniella mitovirus S1	GMV-S1	AAN05635.1
Giardia lamblia virus	GLV	AAB01579.1
Helminthosporium victoriae virus 190S	Hv190S	AAB94791.2
Helicobasidium mompa endornavirus 1	HmEV1-670	BAE94538.1
Helicobasidium mompa mitovirus 1-18	HMV-1	BAD72871.1
Helicobasidium mompa partitivirus V1-1	HmV-V1-1	BAD32677.1
Helicobasidium mompa totivirus 1-17	HmTV1-17	BAC81754.1
Helminthosporium victoriae 145S virus	Hv145S	YP_052858.1
Hepatitis GB virus B	GBV-B	Q69422.1
Human picobirnavirus Hy005102	Hu_PBV-H	BAD98236.1
Human picobirnavirus	Hu_PBV	AAG53584.1
Leishmania RNA virus 1 - 1	LRV-1-1	AAB50024.1
Leishmania RNA virus 2 - 1	LRV-2-1	AAB50031.1
Magnaporthe oryzae virus 2	MoV2	YP_001649206.1
Magnaporthe oryzae virus 1	MoV1	BAD60833.1
Magnaporthe oryzae chrysovrus 1 strain A	MoCV1-A	YP_003858286.1
Mycovirus FusoV	FsV1	NP_624350.1
Ophiostoma mitovirus 1a	OMV-1a	CAJ32466.1
Ophiostoma mitovirus 3a	OMV-3a	NP_660176.1
Ophiostoma mitovirus 4	OMV-4	NP_660179.1
Ophiostoma mitovirus 5	OMV-5	NP_660180.1
Ophiostoma mitovirus 6	OMV-6	NP_660181.1
Oryza sativa endornavirus	OsEV-Nip	BAA06862.1
Otarine picobirnavirus	OtaPBV	JQ776552.1

Persimmon cryptic virus	PeCV	YP_006390091.1
Phaseolus vulgaris endornavirus 1	PvEV-1	BAM68539.1
Phaseolus vulgaris endornavirus 2	PvEV-2	BAM68540.1
Phomopsis vexans RNA virus	PvRV	YP_009115492.1
Phytophthora endornavirus 1	PEV1-OR	CAI47561.1
Picobirnavirus bovine/RUBV-P/IND/2005	BoPBV	ACT64131.1
Picobirnavirus cow/tottori7944/Jap/2013	CowPBV	AB828072.1
Picobirnavirus TK/MN/2011	TKPBV	KJ495690.1
Picobirnavirus monkey/CHN-14/2002	MonPBV	JQ710506.1
Picobirnavirus mouse/M-58/USA/2008	MouPBV	JF755419.1
Enterobacteria phage MS2	MS2	ACY07217.1
Porcine picobirnavirus pig/SD	PorcPBVp	ADO22678.1
Pyrus pyrifolia cryptic virus	PpCV	BAA34783.1
Rose cryptic virus 1	RoCV1	YP_001686786.1
Rosellinia necatrix partitivirus 1-W8	RnV1-W8	YP_392480.1
Rosellinia necatrix victorivirus 1	RnVV1	YP_008130308.1
Saccharomyces 20S RNA narnavirus	ScNV-20S	AAC98925.1
Saccharomyces 23S RNA narnavirus	ScNV-23S	AAC98708.1
Saccharomyces cerevisiae virus L-A	ScV-L-A	AAA50508.1
Saccharomyces cerevisiae virus L-BC	ScV-L-BC	AAB02146.1
Sphaeropsis sapinea RNA virus 1	SsRV1	AAD11601.1
Sphaeropsis sapinea RNA virus 2	SsRV2	AAD11603.1
Tamana bat virus	TABV	AF285080
Trichomonas vaginalis virus 1	TVV1	AAA62868.1
Trichomonas vaginalis virus 2	TVV2	AAF29445.1
Trichomonas vaginalis virus 3	TVV3	AAL37370.1
Tuber excavatum mitovirus	TeMV	AEP83726.1
Ustilaginoidea virens RNA virus 1	UvRV1	YP_007761589.1
Ustilaginoidea virens RNA virus 3	UvRV3	YP_009004156.1
Ustilago maydis virus H1	UmV-H1	AAA81884.1
Vicia faba endornavirus	VfEV-447	CAA04392.1
White clover cryptic virus 1	WCCV1	AAU14888.1

Figure legends

Figure S1: Agarose gel (1%) electrophoresis of PCR-amplified cDNA after FLDS from *M. oryzae* strain S-0412-II. DNA after 25 cycles of PCR was visualized with ethidium bromide. Template cDNA was prepared with (lane 1) or without (lane 2) dsRNA-specific digestion by ShortCut RNaseIII (NEB).

Figure S2: Coverage of sequence reads for each dsRNA segment of MoCV1-A. Sequence coverage for each position along the genome segments is shown in black (left axis). GC skew of the MoCV1-A genome is shown in grey (right axis).

Figure S3: Agarose gel (1%) electrophoresis of the PCR-amplified cDNA after FLDS from the diatom colony. DNA after 40 cycles of PCR was visualized with ethidium bromide. Template cDNA was prepared with (lane 1) or without (lane 2) dsRNA-specific digestion by ShortCut RNaseIII (NEB).

Figure S4: Phylogenetic analysis of RNA replicases (RdRp) found in dsRNA contigs based on amino acid residues. Phylogenetic trees were calculated using MrBayes 3.2.3 and visualized using FigTree 1.4.2 software. The number above each branch refers to the Bayesian posterior probability. The scale bars represent substitutions per site. (A) Phylogenetic tree of the RdRp sequences encoded by 13 *Totiviridae*-related DCADSRVs and 24 selected representative viruses of the family *Totiviridae*. Hv145S was used as an outgroup. (B) Phylogenetic tree of the RdRp sequences encoded by DCADSRV-1 and 12 other selected viruses of the family *Picobirnaviridae*. (C) Phylogenetic tree of the RdRp sequences encoded by DCADSRV-14 and 14 other selected viruses of the family *Partitiviridae*. HvV145S was used as an outgroup. (D) Phylogenetic unrooted tree of the RdRp encoded by DCASSRV-1 and 48 selected viruses of the family *Flaviviridae*. (E) Phylogenetic trees of the RdRp sequences encoded by DCASSRV-2 and six other selected viruses infecting fungi or

oomycetes of the family *Narnaviridae*. MS2 was used as an outgroup. Abbreviations and accession numbers of virus names are listed in Table S5.

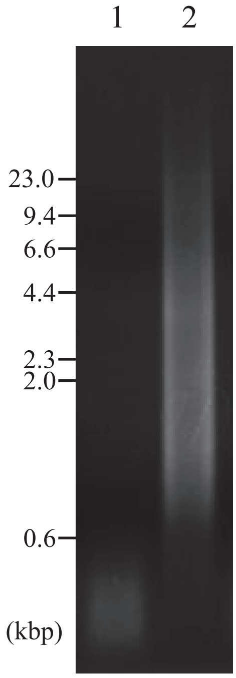

Figure S1
Syun-ichi Urayama

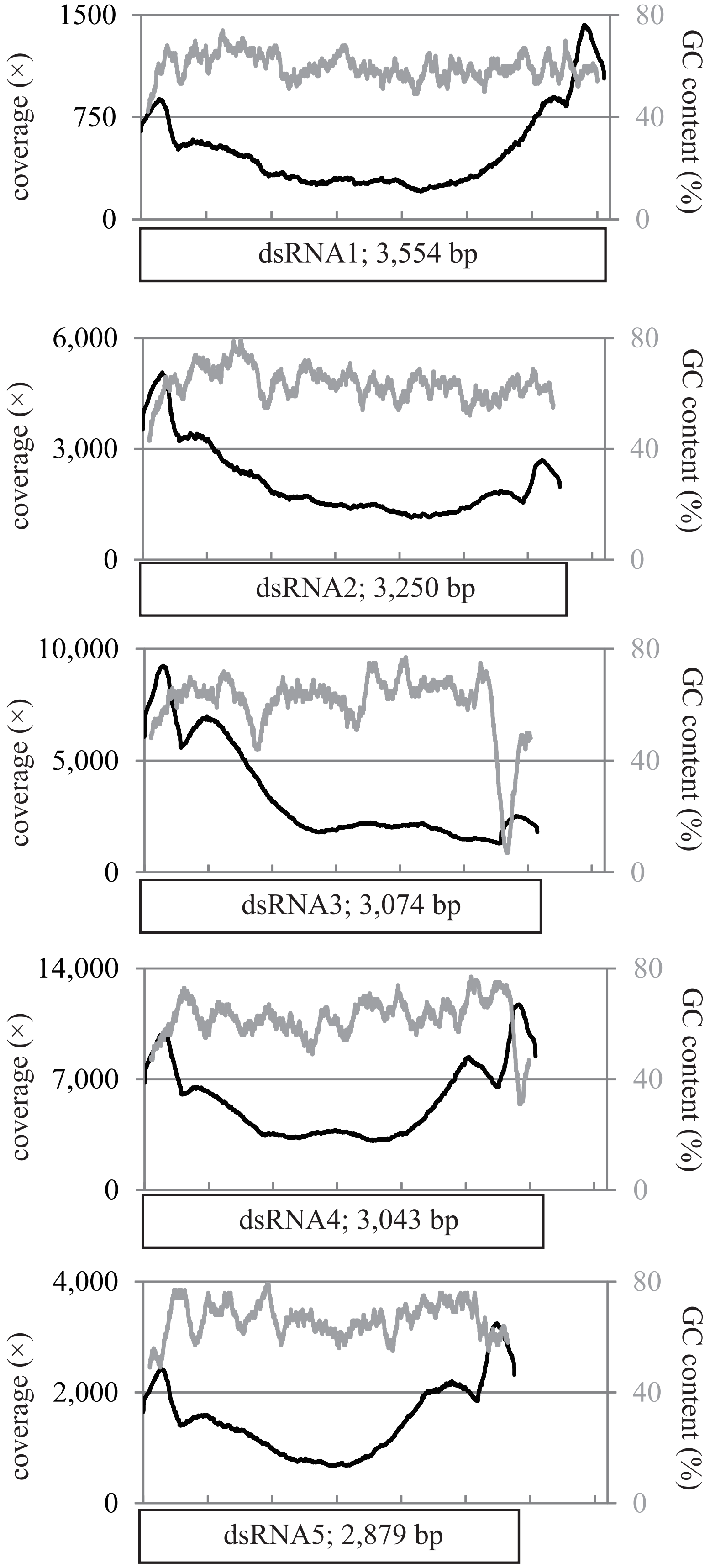

Figure S2

Syun-ichi Urayama

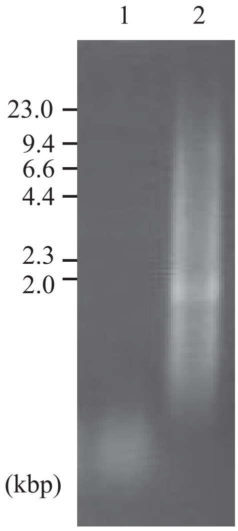

Figure S3

Syun-ichi Urayama

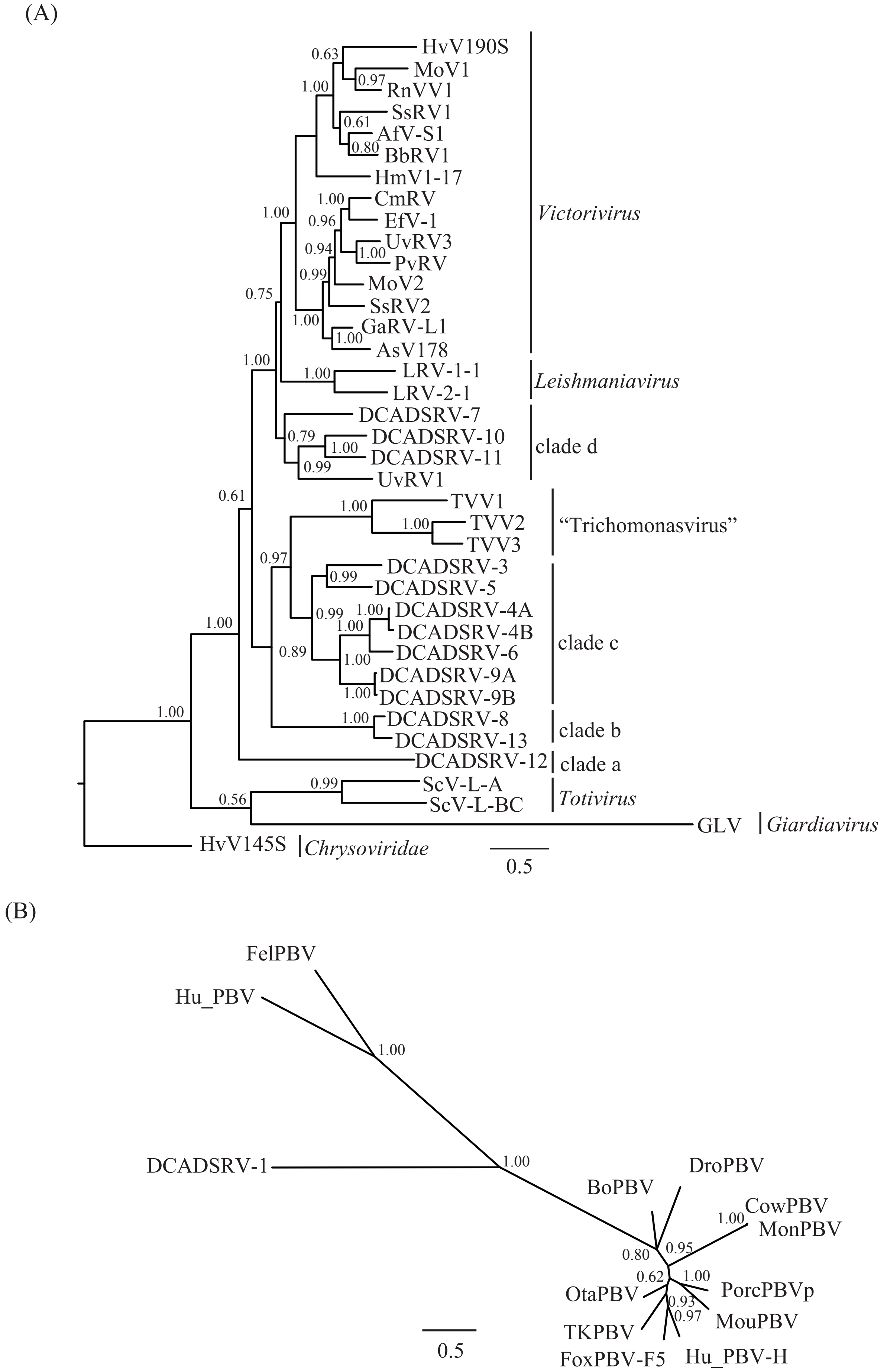

Figure S4

Syun-ichi Urayama

(C)

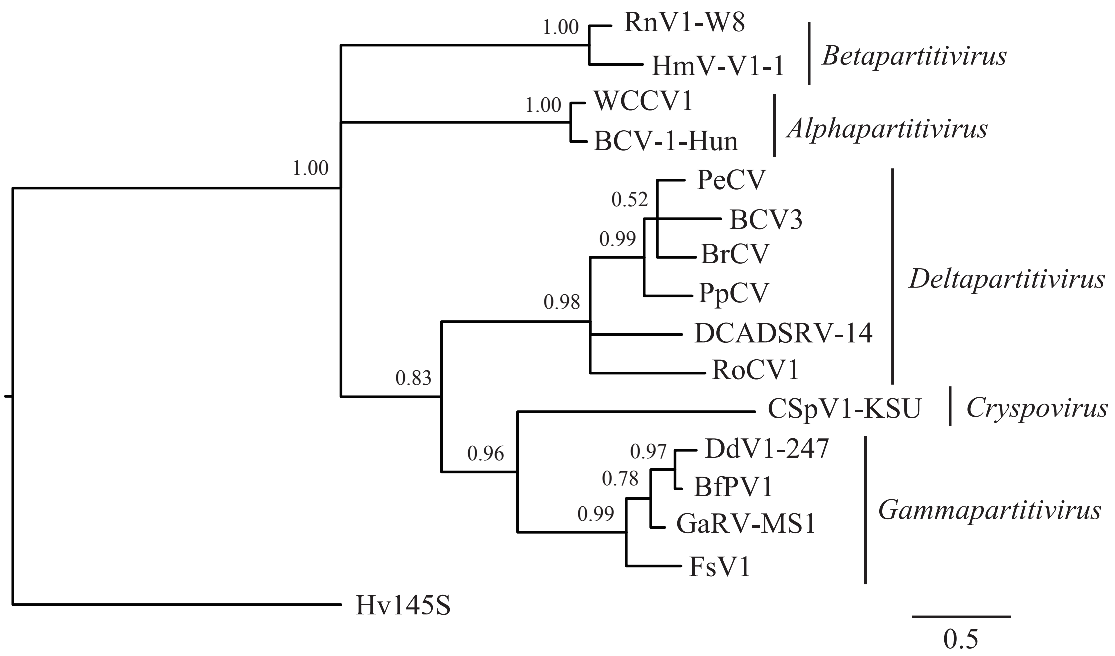

(D)

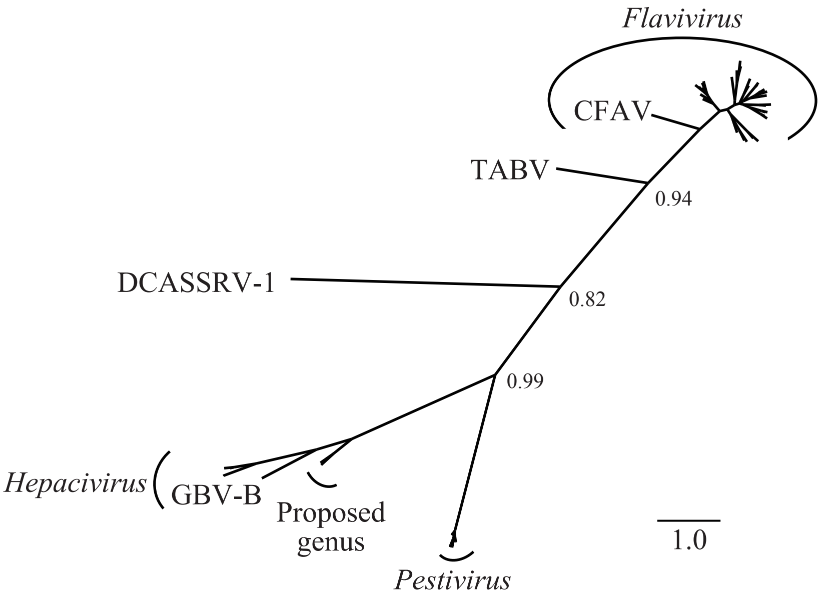

Figure S4

Syun-ichi Urayama

(E)

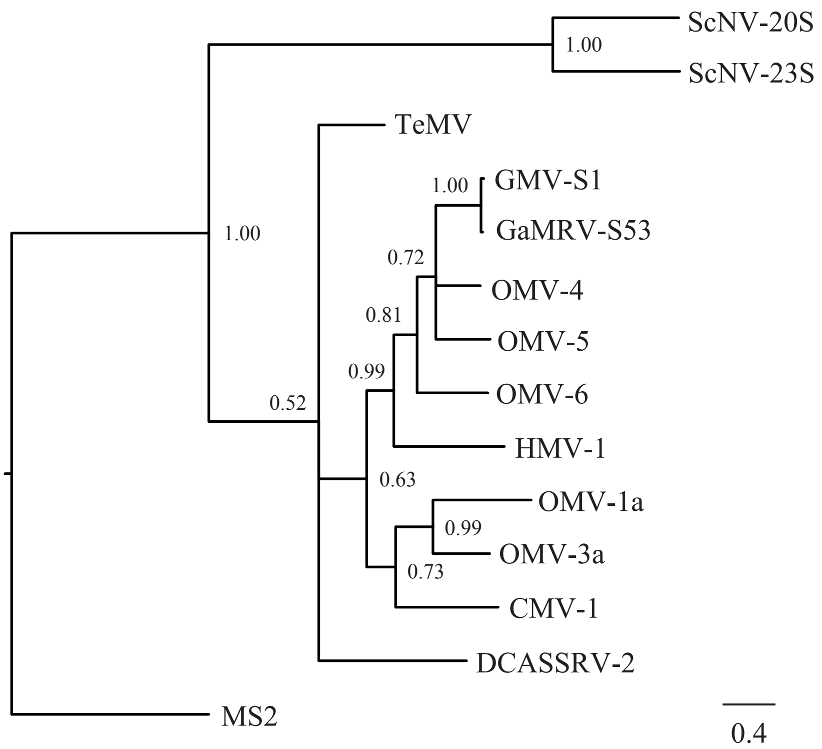

Figure S4

Syun-ichi Urayama
